# Mitochondrial Imaging and Transcriptome Analysis of Bone Mesenchymal Stem Cells During Osteogenesis Under Different Culture Conditions

**DOI:** 10.3390/biom15111623

**Published:** 2025-11-19

**Authors:** Qicheng Li, Tianze Sun, Shiyan Liu, Lu Zhang, Yuhui Kou

**Affiliations:** 1 Department of Trauma and Orthopedics, Peking University People’s Hospital, Beijing 100044, China; qicheng.li@pku.edu.cn (Q.L.); 2411110398@stu.pku.edu.cn (T.S.); 2 Key Laboratory of Trauma and Neural Regeneration, Peking University, Beijing 100044, China; 3 Department of Physiology, School of Basic Medical Sciences, Shenzhen University, Shenzhen 518060, China; 2100243061@email.szu.edu.cn (S.L.); 2300243050@email.szu.edu.cn (L.Z.); 4 National Center for Trauma Medicine, Beijing 100044, China

**Keywords:** bioinformatics, bone mesenchymal stem cells, mitochondrial imaging, osteogenic differentiation, RNA-Seq, three-dimensional culture

## Abstract

Bone mesenchymal stem cells (BMSCs) are multipotent progenitors with significant potential for bone tissue engineering and regenerative medicine. This study compared the mitochondrial imaging and transcriptome of BMSCs under two-dimensional (2D) and three-dimensional (3D) culture conditions during osteogenesis. 2D BMSCs were induced toward osteogenesis for 7, 14, and 21 days, while 3D BMSCs were induced for 21 days. Osteogenic mineralization was assessed by Alizarin Red S (ARS) staining, and whole-transcriptome sequencing (RNA-Seq) was performed to elucidate gene expression profiles. Furthermore, mitochondrial morphology in live cells was monitored at 0, 7, 14, and 21 days of 2D osteogenic differentiation to observe the mitochondrial changes. High-Sensitivity Structured Illumination Microscopy (HIS-SIM) imaging showed that mitochondrial morphology in BMSCs underwent a shift toward elongated and interconnected networks over time. The transcriptional profile showed that genes associated with skeletal morphogenesis, bone development, and extracellular matrix organization were significantly upregulated in 3D culture systems. These findings indicate that 3D culture is associated with a transcriptional profile enriched in pathways commonly observed during in vivo osteogenesis, which can inform scaffold-based bone-regeneration strategies.

## 1. Introduction

Bone mesenchymal stem cells (BMSCs) are multipotent stromal cells with osteogenic, chondrogenic, and other mesenchymal differentiation capacities, together with a high proliferative potential [1,2]. These characteristics make BMSCs a key cellular resource for bone tissue engineering. However, translating their inherent differentiation potential into predictable bone formation remains challenge. The challenging aspect of osteogenic regulation is mostly related to the dynamic interactions between extracellular matrix (ECM) signaling [3], cell–cell crosstalk [4], and metabolic reprogramming [5]. This is something that traditional detection methods cannot completely elucidate. A broader and more systematic understanding of how the cultural microenvironment influence lineage decisions could lead to the rational design of scaffold materials and induction protocols that better mimic in vivo bone development.

Traditional two-dimensional (2D) monolayer culture offers experimental convenience but imposes artificial geometry, alters mechanical transduction, and induces aberrant diffusion kinetics, thereby distorting cell fate determination and extracellular matrix deposition. In contrast, three-dimensional (3D) environments provide cells with tissue-like structure and viscoelastic microenvironment [6] that influence integrin binding [7], cytoskeletal tension [8]. Although previous studies indicate that 3D conditions can enhance osteogenic outcomes [9,10], the comprehensive transcriptional changes and their synergistic mechanisms triggered by difference between 3D and 2D cultures remain incompletely elucidated during the osteogenesis of BMSCs. This gap limits our ability to link functional improvements to specific pathways or regulatory networks.

High-throughput RNA sequencing (RNA-Seq) offers an unbiased approach to explore the genome-wide expression programs underlying lineage differentiation, particularly for analyzing expression patterns governing skeletal development, matrix organization, and mineralization processes. Concurrently, mitochondrial function has emerged as a determinant, rather than a passive consequence [11]. During osteogenic differentiation, bioenergetics, redox balance, and organelle dynamics are coupled with transcriptional regulation. Therefore, establishing an integrated framework that links cellular metabolic states to gene regulatory outputs governing bone formation can be achieved by longitudinally assessing mitochondrial activity during 2D induction and integrating this with endpoint transcriptomic profiling under both 2D and 3D culture conditions.

In this study, we compared rat BMSCs undergoing osteogenic differentiation under 2D conditions (for 7, 14, and 21 days) and 3D conditions (for 21 days). We performed ARS staining to assess mineralization, measured mitochondrial morphology in live cells at specific intervals, and conducted whole-transcriptome RNA sequencing (RNA-Seq). We postulated that BMSCs cultured as 3D spheroids would exhibit transcriptional profiles more closely aligned with in vivo osteogenesis, particularly in pathways related to extracellular matrix (ECM) assembly, osteoblast differentiation, and skeletal morphogenesis. By identifying the distinct molecular and metabolic signatures of osteogenesis in 3D vs. Two-dimensional cultures, our findings offer mechanistic insights that can inform the optimization of scaffold-based bone-regeneration strategies.

## 2. Materials and Methods

### 2.1. Isolation, Culture, and Osteogenic Differentiation of BMSCs

Bone mesenchymal stem cells (BMSCs) were isolated from 3-day-old Sprague-Dawley rats (Vital River Laboratory Animal Technology Co., Ltd., Beijing, China). After euthanasia, the femur and tibia were aseptically dissected. The epiphyses were removed, and the bone marrow was flushed out with α-MEM (Hyclone, Logan, UT, USA). The marrow suspension was centrifuged at 1500 rpm for 3 min. The pellet was then resuspended in complete culture medium consisting of α-MEM supplemented with 10% FBS (Gibco, Gaithersburg, MD, USA) and 1% penicillin-streptomycin (Hyclone, Logan, UT, USA). Cells were seeded into T25 flasks (Corning, NY, USA) and incubated without disturbance for 48 h. Subsequently, a half-medium change was performed to remove non-adherent cells. Upon reaching 80–90% confluence, the adherent cells were harvested using 0.25% trypsin (Hyclone, Logan, UT, USA) and subcultured at appropriate dilutions. Cells from passages 2–4 were used for all subsequent experiments. To induce osteogenic differentiation in two-dimensional (2D) culture system, BMSCs were plated at a density of 2 × 10^5^ cells per well in 6-well plates (Corning, NY, USA). Once the cells reached 90% confluence, the growth medium was replaced with osteogenic induction medium consisting of α-MEM, 10% FBS, 10 mM β-glycerophosphate (MCE, Monmouth Junction, NJ, USA), 50 µg/mL L-ascorbic acid 2-phosphate (MCE, Monmouth Junction, NJ, USA), and 100 nM dexamethasone (MCE, Monmouth Junction, NJ, USA). The cultures were maintained at 37 °C in a humidified atmosphere of 5% CO_2_.

### 2.2. Generation of BMSCs Spheroid and Osteogenic Differentiation

To generate BMSCs spheroids and induce osteogenic differentiation under three-dimensional (3D) culture conditions, BMSCs suspension was seeded into specialized ultra-low attachment Honeycomb Chips (VIVOID Biotechnology, Suzhou, China). The chips were incubated at 37 °C in a humid atmosphere of 5% CO_2_ for 48 h to facilitate spheroid formation. The BMSCs spheroids were observed and photographed using a light microscope (Nikon, Tokyo, Japan), and their diameters were subsequently analyzed using ImageJ software (version 1.54p). Following incubation, the wells were carefully rinsed with PBS (Hyclone, Logan, UT, USA) to remove residual growth medium. Subsequently, osteogenic induction medium was added to each well to initiate differentiation. The spheroids were cultured for additional 21 days under these standard conditions. The medium was replaced every 72 h throughout the differentiation period to maintain nutrient and inductive factor levels.

### 2.3. Alizarin Red S Staining

The osteogenic differentiation of BMSCs was assessed by Alizarin Red S staining (Beyotime Biotechnology, Shanghai, China). For 2D cultures, cells were fixed with 4% paraformaldehyde (PFA, Solarbio, Beijing, China) for 30 min at room temperature, rinsed with PBS, and subsequently incubated with ARS for 30 min. Following incubation, unbound dye was removed by washing with PBS. Three-dimensional BMSCs spheroids were stained with ARS following the same protocol. Mineralized matrix deposition in both 2D and 3D cultures was then evaluated by light microscopy.

### 2.4. Mitochondrial Imaging of Differentiated BMSCs

BMSCs were plated on 35 mm glass-bottom dishes (NEST, Wuxi, China) with PDL (Solarbio, Beijing, China) and cultured under standard conditions. To visualize mitochondria, the cells were incubated with 250 nM PK Mito Red (GENVIVO, Nanjing, China) in α-MEM for 15 min at 37 °C. After staining, the cells were washed twice with α-MEM to eliminate unbound dye. Subsequently, 2 mL of fresh α-MEM was added, and live-cell imaging was performed using High-Sensitivity Structured Illumination Microscopy (HIS-SIM). Mitochondrial morphology from three independent cultures, including Branches, Branch End Points, and Mean Branch Length, was quantitatively assessed for the acquired images using the Mitochondria Analyzer plugin in ImageJ.

### 2.5. RNA-Seq, Library Construction

Total RNA was isolated from 2D and 3D BMSCs using TRIzol-Chloroform extraction and isopropanol precipitation. Concentration and integrity of RNA were determined using the Qubit Fluorometer (Invitrogen, Carlsbad, CA, USA) and Bioanalyzer 2100 System (Agilent Technologies, Santa Clara, CA, USA). Sequencing libraries were constructed from 1 µg of RNA using NEBNext^®^ UltraTM RNA Library Prep Kit for Illumina^®^ (NEB, Ipswich, MA, USA). mRNA was enriched from the total RNA using oligo (dT) magnetic beads and subsequently fragmented by incubation in divalent cations at elevated temperature. First-strand cDNA synthesis was performed using random hexamer primers and M-MuLV Reverse Transcriptase. The second strand was then synthesized using DNA Polymerase I and RNase H. The cDNA fragments were end-repaired to generate blunt ends, adenylated at the 3′ ends, and ligated to NEBNext hairpin adapters. cDNA fragments of 250–300 bp were size-selected using AMPure XP system (Beckman Coulter, Beverly, MA, USA). The adaptor loops were then cleaved with USER enzyme (37 °C, 15 min; 95 °C, 5 min), and the libraries were amplified by limited-cycle PCR with Phusion High-Fidelity DNA polymerase and index primers. The final cDNA libraries were purified with AMPure XP system, and their quality was verified using an Agilent Bioanalyzer 2100.

### 2.6. Data Analysis

Sequencing was performed on an Illumina Novaseq platform (Illumina, San Diego, CA, USA) and 150 bp paired-reads were generated. Raw FASTQ files were processed using in-house scripts to remove adapter-contaminated, poly-N, and low-quality reads, yielding clean reads. The Q20, Q30, and GC content of the clean reads were calculated. The reference genome and gene annotation were downloaded from public repository. These files were then indexed using HISAT2. Subsequently, the paired-end clean reads were aligned to the reference genome with HISAT2 in a splice-aware mode, utilizing the provided annotation for guidance. Gene-level read counts were generated using featureCounts. To quantify gene expression abundance, we calculated the Transcript Per Million mapped reads (TPM). We performed principal component analysis (PCA) on the log_2_(TPM + 1) transformed matrix to evaluate sample clustering and identify potential batch effects. All subsequent analyses were conducted using these high-quality, aligned datasets.

### 2.7. DEGs Analysis and GO, KEGG Analysis

Differential gene expression (DEG) analysis was performed on two groups, each with three biological replicates, using DESeq2 (version 0.11.5) for data normalization and statistical analysis [12]. DESeq2 uses a negative binomial distribution model, and genes with an adjusted *p*-value < 0.05 were considered differentially expressed. Gene Ontology (GO) enrichment was performed with the clusterProfiler R package (version 4.12.6), considering GO terms with a corrected *p*-value < 0.05 as significantly enriched [13]. KEGG pathway enrichment was also analyzed using clusterProfiler, highlighting the involvement of differentially expressed genes in specific biological pathways.

### 2.8. Time-Series Analysis of Differential Gene Expression

Using the R package DESeq2, we identified genes that were significantly differentially expressed compared to the 3D3W group and took their intersection to define time-series genes. We selected time-series genes that exhibited significant expression with an adjusted *p* < 0.05 in DESeq2 analyses. The identified time-series genes were clustered using the R package Mfuzz (2.64.0) that performs soft clustering based on the fuzzy c-means algorithm. Average TPM values (three samples per group) of individual genes were employed as input values for Mfuzz clustering. The number of clusters was set to 8 and the fuzzifier coefficient, M, to 2.58.

### 2.9. Visualization of GO Gene Sets

The significant pathways associated with GO terms of interest were visualized to summarize expression patterns and gene-function relationships. Visualization is achieved through the enrichplot package. For this analysis, we focused on significant GO pathways related to osteogenesis, angiogenesis, and immunomodulation. The osteogenesis pathways included “bone development” and “osteoblast differentiation”. The angiogenesis pathways included “endothelial cell chemotaxis” and “regulation of endothelial cell chemotaxis”. The immunomodulation pathways included “leukocyte aggregation” and “positive regulation of inflammatory response”. These pathways were selected based on their relevance to the biological processes of interest in our study.

### 2.10. Protein–Protein Interaction (PPI) Network

In order to further explore the interaction relationships among the genes within the enriched GO pathways, we constructed protein interaction networks for the target gene sets. The official gene symbols were submitted to the STRING system (for *Rattus norvegicus*) to query physical and functional associations [14]. The STRING interaction table was imported into Cytoscape (version 3.10.3) for network analysis and chart creation, and the corresponding enriched pathways were annotated with the color of the outer ring of the points.

### 2.11. Statistical Analysis

All statistical analyses were conducted using GraphPad Prism 8.0. Results are presented as mean ± SD. Differences between two independent groups were assessed using a two-tailed, unpaired Student’s *t* test. All analyses were two-sided, and a *p*-value below 0.05 was deemed to indicate statistical significance.

## 3. Results

### 3.1. BMSCs Spheroid Formation in Honeycomb Chips

**Figure 1:**
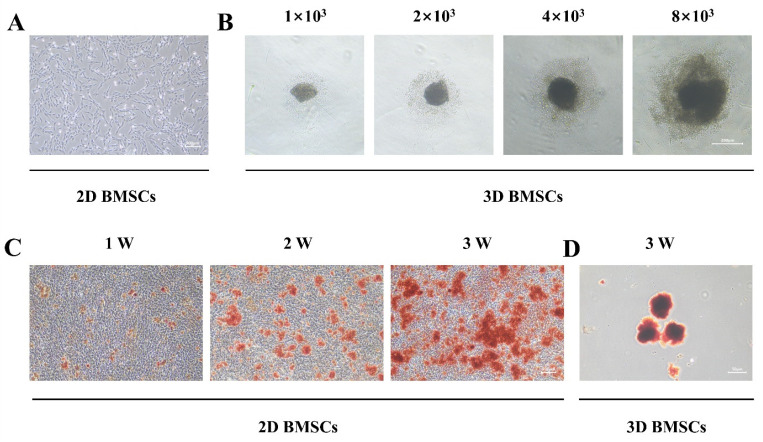
Culture and osteogenic differentiation of BMSCs. (**A**) Culture of 2D BMSCs. Scale bar = 200 μm; (**B**) 3D BMSCs spheroid formation with different density. Scale bar = 200 μm; (**C**) ARS staining after 1 week, 2 weeks, and 3 weeks of osteogenic differentiation of 2D BMSCs. Scale bar = 200 μm; (**D**) ARS staining after 3 weeks of osteogenic differentiation of 3D BMSCs. Scale bar = 50 μm.

BMSCs were isolated from rat bone marrow using the whole bone marrow adherence method [15]. Figure 1A illustrated the morphological characteristics of BMSCs, which typically exhibited a fibroblast-like, spindle-shaped appearance. Cells from passages 2–4 were seeded into U-bottomed ultra-low attachment honeycomb chips at densities of 1 × 10^3^, 2 × 10^3^, 4 × 10^3^, and 8 × 10^3^ cells per well to facilitate spheroid formation. Following 48 h culture period, the formation of BMSCs spheroids was observed, and the diameter of the spheroids increased in a cell density-dependent manner. Specifically, spheroids formed at higher seeding densities (4 × 10^3^ and 8 × 10^3^ cells per well) exhibited larger diameters compared to those formed at lower densities (1 × 10^3^ and 2 × 10^3^ cells per well), as shown in Figure 1B.

### 3.2. Osteogenic Differentiation of BMSCs in 2D and 3D Culture Conditions

The osteogenic differentiation of BMSCs was assessed under different culture conditions. In the 2D culture system, BMSCs were cultured in 6-well plates for 1, 2, and 3 weeks, and osteogenic differentiation was evaluated by ARS staining to visualize calcium deposition. The number of calcium nodules increased progressively with the duration of osteogenic induction, indicating that the osteogenic potential of BMSCs enhanced over time in the 2D culture system (Figure 1C). In the 3D culture system, BMSCs maintained for 3 weeks exhibited a more pronounced osteogenic phenotype (Figure 1D). These characteristics likely integrate both the influence of the 3D microenvironment and the mature developmental state of BMSCs.

### 3.3. Changes in Mitochondrial Morphology During Osteogenic Differentiation

**Figure 2:**
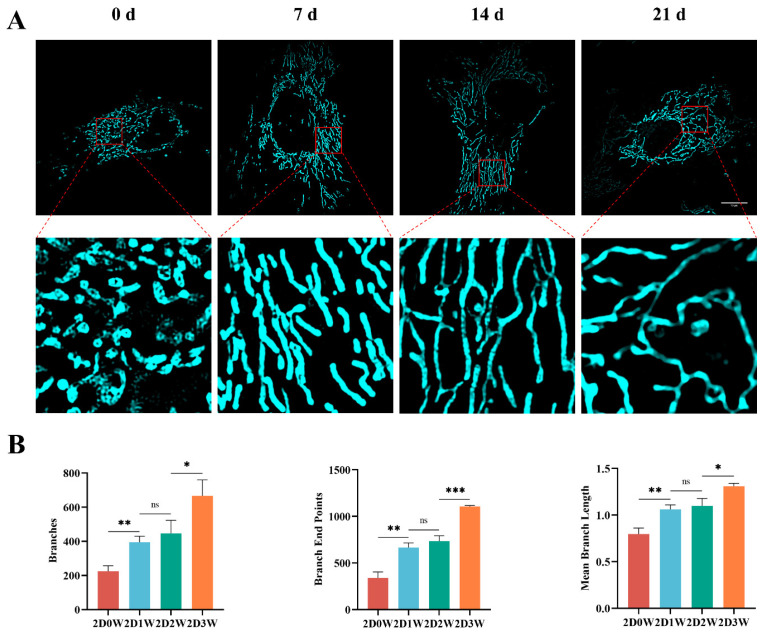
Mitochondrial changes in BMSCs during osteogenic differentiation. (**A**) Representative images of PK Mito Red-stained mitochondria in BMSCs. Scale bar = 10 μm; (**B**) Quantitative analysis of morphological changes in mitochondria at different time points (*n* = 3, mean ± SD). ns, no significant; * *p* < 0.05; ** *p* < 0.01; *** *p* < 0.001.

We evaluated the mitochondrial morphology of BMSCs on days 0, 7, 14, and 21 post-osteogenic induction under 2D culture conditions (Supplementary Video). The differentiation process caused changes in mitochondrial morphology that are consistent with previous reports of mitochondrial dynamics contributing to differentiation. At day 0, mitochondria predominantly displayed a fragmented and spherical morphology. By day 7, the mitochondria had elongated and begun to form interconnected networks. This trend continued through day 14, with a notable increase in network complexity and branching. By day 21, the mitochondrial networks were extensively branched, exhibiting significant increases in number of branches, branch endpoints, and the mean branch length compared to earlier time points (Figure 2A,B). In 2D culture system, the observed progressive changes in mitochondrial architecture appear to correlate with stages of osteogenic differentiation, potentially indicating a role for mitochondrial dynamics in the transition of BMSCs toward an osteoblast-like phenotype. The extensive branching and elongation observed in the later stages could be consistent with an increase in energy metabolism and biosynthetic demands during bone matrix formation.

### 3.4. Gene Expression Profiles During Osteogenic Differentiation of BMSCs

**Figure 3:**
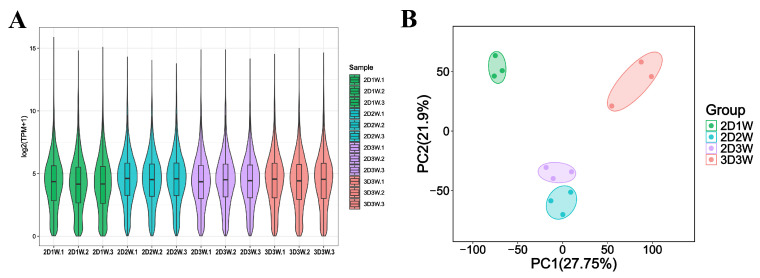
The quality of RNA-seq data from each BMSCs sample. (**A**) The distribution of gene expression levels in each BMSC sample; (**B**) Two-dimensional scatter plot principal components.

We performed RNA sequencing to elucidate the molecular mechanisms driving the osteogenic differentiation of BMSCs under 2D or 3D culture conditions. We extracted total RNA from BMSCs that underwent osteogenic induction for 2D at week 1, week 2, and week 3 (2D1W, 2D2W and 2D3W) and 3D at week 3 (3D3W). The samples used in the analysis obtained about 40~ million reads per sample. Each condition was processed in three biological replicates. The raw sequencing reads were aligned to the *Rattus norvegicus* reference genome Rnor_6.0 which yielded about 30~ million uniquely mapped reads per sample (Figure 3A). Principal Component Analysis (PCA) revealed that the first principal component (PC1) accounted for around 27.75% and second principal component (PC2) contributed 21.9% of the variance as seen in Figure 3B. These findings show distinct gene expression patterns in different time points and culture conditions, providing a comprehensive overview of the molecular changes occurring during the osteogenic differentiation of BMSCs.

### 3.5. Identification of DEGs During Osteogenic Differentiation

**Figure 4:**
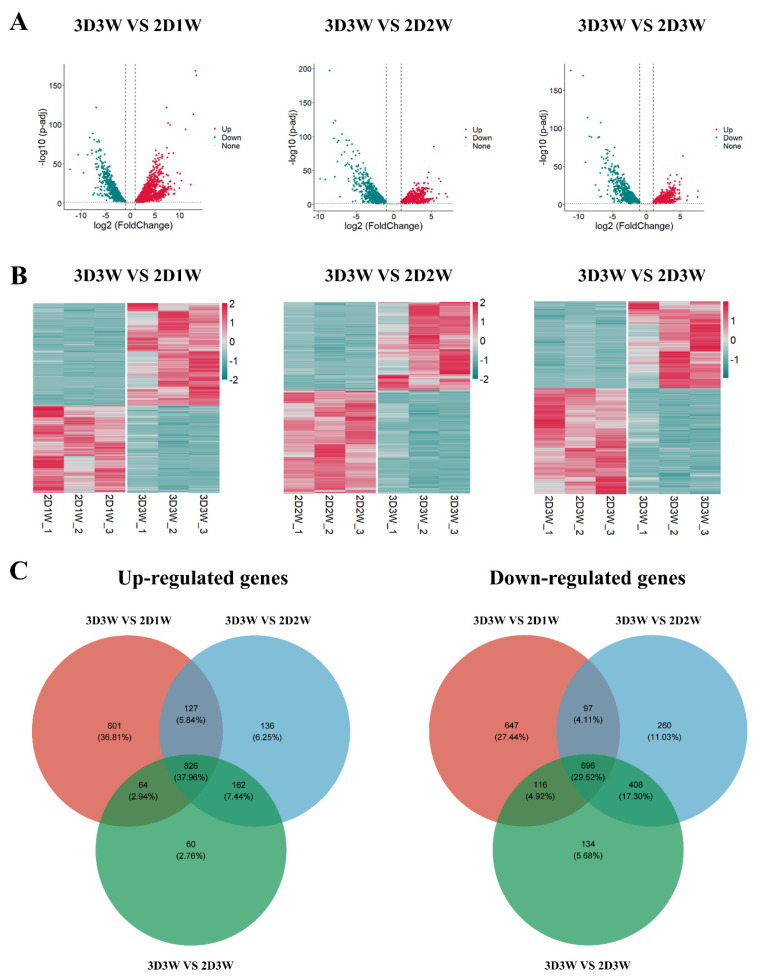
Identification of DEGs in BMSCs under 2D and 3D culture conditions. (**A**) Volcano plots showing up- and down-regulated DEGs at 3D3W vs. 2D1W, 3D3W vs. 2D2W, and 3D3W vs. 2D3W; (**B**) Heatmap of 3D3W vs. 2D1W, 3D3W vs. 2D2W, and 3D3W vs. 2D3W; (**C**) Venn maps of DEGs under 2D and 3D culture conditions, with co-upregulated genes on the left and co-downregulated on the right.

We performed transcriptomic analysis to identify DEGs during osteogenic differentiation. Initially, volcano plots were used to visualize the distribution of DEGs between the different groups. The comparison of 3D3W vs. 2D1W revealed 1818 upregulated and 1556 downregulated genes. Similarly, the 3D3W vs. 2D2W comparison exhibited 1251 upregulated and 1461 downregulated genes, while 3D3W vs. 2D3W showed 1112 upregulated and 1354 downregulated genes (Figure 4A). Comparisons between 3D3W and 2D1W, as well as 3D3W and 2D2W, revealed relatively higher numbers of DEGs. This may stem from both the less mature 2D osteogenic differentiation process and the unique 3D microenvironment. Further analysis using heatmaps for 3D3W vs. 2D1W, 3D3W vs. 2D2W, and 3D3W vs. 2D3W clearly demonstrated the distribution of the DEGs across these comparisons with distinct clustering patterns for upregulated and downregulated genes (Figure 4B). Table 1 listed the top five most significantly upregulated and downregulated genes for each comparison, providing a focused view of key molecular regulators in the osteogenic process. Additionally, Venn diagram analysis identified 826 commonly upregulated genes and 696 commonly downregulated genes across the three comparisons (3D3W vs. 2D1W, 3D3W vs. 2D2W, and 3D3W vs. 2D3W) (Figure 4C). These comparisons should be regarded as composite markers reflecting the combined effects of dimension and maturation, rather than purely dimension-specific effects. The above analysis has laid an important foundation for us to identify the key regulatory genes and related to the temporal dynamics of bone formation and differentiation.

### 3.6. KEGG and GO Analysis of DEGs

**Figure 5:**
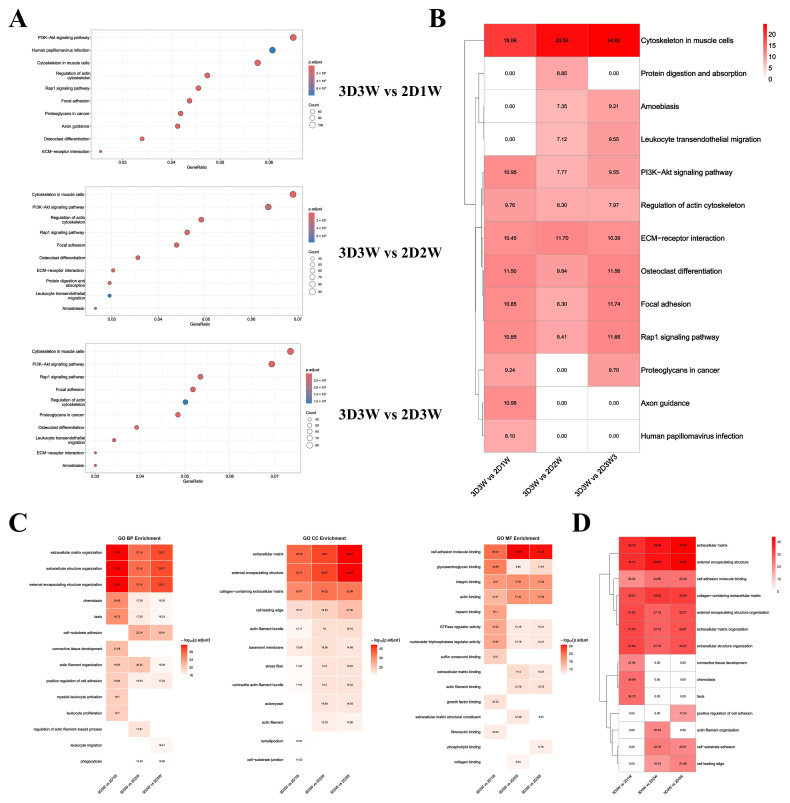
KEGG and GO analysis of DEGs. (**A**) Bubble charts showing pathways with *p* < 0.01 at 3D3W vs. 2D1W, 3D3W vs. 2D2W, and 3D3W vs. 2D3W; (**B**) Clustering the KEGG analysis results at three comparisons; (**C**) Clustering of enriched BP, CC, and MF terms at 3D3W vs. 2D1W, 3D3W vs. 2D2W, and 3D3W vs. 2D3W; (**D**) Heatmap of the top ten pathways for the GO enrichment results of the differences between 3D3W and 2D1W, 3D3W and 2D2W, and 3D3W and 2D3W.

To identify pathways and biological functions linked to the observed changes, we performed KEGG and GO enrichment analysis on the DEGs from the following comparisons: 3D3W vs. 2D1W, 3D3W vs. 2D2W, and 3D3W vs. 2D3W. The KEGG analysis identified significantly enriched pathways (*p* < 0.01) across all comparisons. The most pronounced clustering of these pathways was consistently observed in the 3D3W vs. 2D1W, 3D3W vs. 2D2W, and 3D3W vs. 2D3W (Figure 5A). Notably, pathways related to the cytoskeleton in muscle cells, PI3K-Akt signaling pathway, regulation of actin cytoskeleton, and ECM-receptor interaction were most significantly enriched, suggesting their pivotal roles in the temporal progression under our experimental conditions (Figure 5B).

We also conducted GO enrichment analysis on the DEGs, which revealed several biological processes that were highly conserved across the three comparisons. Figure 5C displays the top 10 enriched GO terms for each comparison, showing similarities among 3D3W vs. 2D1W, 3D3W vs. 2D2W, and 3D3W vs. 2D3W groups. A key finding was the strong association with extracellular matrix (ECM) secretion, indicating that ECM-related processes contribute to in the observed differential gene expression. To further elucidate the functional roles of these pathways, we have identified the overall pathway with the most significant differences among the three sets of GO enrichment results. The results revealed that the top 10 clusters in each group were predominantly enriched for terms related to the extracellular matrix (GO:0031012), external encapsulating structure (GO:0030312), cell adhesion molecule binding (GO:0050839), collagen-containing extracellular matrix (GO:0062023), external encapsulating structure organization (GO:0045229), extracellular matrix organization (GO:0030198), extracellular matrix organization (GO:0030198) (Figure 5D). These findings underscore the importance of ECM-related processes in the biological responses to the experimental conditions, particularly in terms of structural organization and cell–matrix interactions.

### 3.7. Time-Series Analysis of Commonly up- and Down-Regulated DEGs

**Figure 6:**
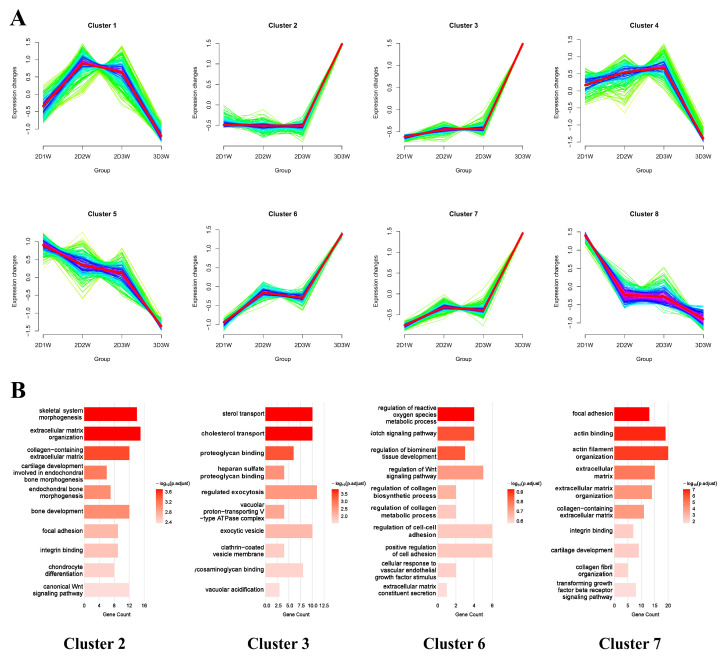
Time-series analysis of DEGs. (**A**) Clustering analysis of time-series genes during osteogenic differentiation using Mfuzz; (**B**) GO enrichment analysis of cluster 2, 3, 6 and 7.

To delineate the temporal transcriptional programs during osteogenic induction, we performed a time-course analysis across the 2D1W, 2D2W, 2D3W and 3D3W. Using DESeq2 with a time-series design, we identified 1522 DEGs. Application of fuzzy c-means clustering (Mfuzz) to the normalized expression trajectories partitioned these DEGs into eight cluster (ranging from 107 to 356 genes, Figure 6A, Table 2). Four clusters (2, 3, 6, and 7), comprising 624 genes, showed a pronounced late-phase upregulation, peaking selectively in 3D3W relative to all 2D time points. We designated this gene set as the “3D-enhanced signatures”. Functional enrichment analysis of these clusters highlighted processes central to osteogenesis and 3D microenvironment sensing, including skeletal system morphogenesis, extracellular-matrix organization, focal adhesion, actin binding, Notch signaling, and metabolic adaptations in cholesterol transport and regulation of reactive oxygen species (Figure 6B). This coordinated upregulation aligns with enhanced matrix deposition, mechanotransductive signaling, and redox homeostasis, which are hallmarks of advanced osteogenic differentiation under 3D conditions.

### 3.8. GO-Enriched Gene Sets and PPI Networks

**Figure 7:**
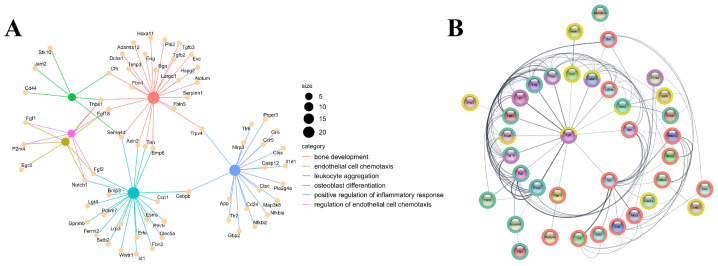
GO-enriched gene sets and PPI networks. (**A**) GO-enriched genes related to osteogenesis, angiogenesis, and immunomodulation; (**B**) PPI networks for genes. Each dot represents a gene, and the thickness of the line represents the interaction intensity. The color of the outer circle corresponds to the enriched pathway: red: “positive regulation of inflammatory response”; Green: “bone development” Yellow: “Osteoblast Differentiation” Purple: “Regulation of endothelial cell chemotaxis”.

After merging the Mfuzz clusters that were upregulated under the 3D condition (clusters 2, 3, 6, and 7), GO enrichment converged on three core processes in bone repair: osteogenesis (“bone development”, “osteoblast differentiation”), angiogenesis (“endothelial cell chemotaxis”, “regulation of endothelial cell chemotaxis”), and immunomodulation (“leukocyte aggregation”, “positive regulation of inflammatory response”). These functional groups are coordinated transcription programs rather than isolated pathways. The concept of osteo-angio-immune coupling is well established and critical for effective skeletal regeneration. We targeted genes involved in two or more functional axes to find pleiotropic regulators. This screening process detected a small number of multifunctional nodes (*CFH*, *THBS1*, *FGF18*, *SEMA4D*, *AXIN2*, *TNN*, *BMP6*, *CEBPB*, *FGF2*, *NOTCH1*, *FGF1*, *P2RX4*, and *TRPV4*) interconnected in networks involving extracellular matrix organization, growth-factor signaling, and inflammatory cell trafficking (Figure 7A). PPI analysis of the merged gene set revealed a densely interconnected subnetwork that couples osteogenic and angiogenic effectors, with immune modulators positioned at the interface at the network. Within this network topology, FGF2 emerged as the central hub (interacting closely with nodes such as THBS1, NOTCH1, ENG, FGF1, TGFB3, HSPG2), suggesting it may play a coordinating role across the three axes (Figure 7B).

## 4. Discussion

Bone mesenchymal stem cells (BMSCs) have emerged as crucial “seed cells” in regenerative medicine due to their multipotency and relative ease of isolation, with demonstrated potential for treating neurological [16], skeletal [17], and muscular disorders [18]. Their therapeutic potential is supported by extensive preclinical and clinical investigation, with 673 studies currently registered on ClinicalTrials.gov. However, the conventional two-dimensional (2D) culture systems used to expand and differentiate BMSCs inadequately recapitulate the complex three-dimensional (3D) microenvironment in vivo, often resulting in compromised differentiation fidelity and regenerative efficacy following transplantation. Additionally, other mesenchymal stem cells (MSCs), such as adipose-derived MSCs, umbilical cord-derived MSCs, and amnion-derived MSCs, exhibited differences in transcriptome, metabolic levels, folding levels, and doubling levels under 3D vs. Two-dimensional culture conditions [19,20,21,22]. Our findings align with the previous studies that 3D culture platforms can better mimic native tissue conditions [23,24]. In our study, 3D BMSCs exhibited a significantly higher capacity for osteogenic mineralization than 2D BMSCs as shown by a significant increase in ARS staining after induction for 21 days. Generally, the 3D microenvironment plays a crucial role in the functional maturation of BMSCs towards the osteogenic lineage.

Mitochondria are increasingly recognized as dynamic organelles that participate in cell fate decisions, far beyond their traditional role as mere energy producers [25,26]. Prior studies have established that mitochondrial dynamics, including fission, fusion, and network remodeling, are integral to the differentiation processes of stem cells [27,28,29]. Furthermore, mitochondrial dysfunction is a key hallmark of cellular senescence, adversely affecting the regenerative potential of BMSCs [30]. In the present study, we observed a progressive reorganization of the mitochondrial network during osteogenic induction in 2D culture. Mitochondria transitioned from a predominantly fragmented morphology at day 0 to a highly elongated and interconnected state with increased branching complexity by day 21. This morphological shift likely reflects an adaptive response to meet the elevated bioenergetic and biosynthetic demands of bone matrix synthesis and mineralization, highlighting the functional importance of mitochondrial dynamics in osteogenic differentiation.

To explore the molecular mechanisms governing osteogenic differentiation of BMSCs, we conducted transcriptomic analysis on cells harvested from the 2D1W, 2D2W, 2D3W, and 3D3W groups. RNA sequencing revealed 4534 DEGs between 3D and 2D culture conditions. Comparisons of 3D3W vs. 2D1W (1818 up/1556 down), 3D3W vs. 2D2W (1251 up/1461 down), and 3D3W vs. 2D3W (1112 up/1354 down) identified 826 consistently up-regulated and 696 consistently down-regulated genes across all three comparisons. Enrichment analysis of the DEGs demonstrated significant enrichment in terms related to extracellular matrix (ECM) organization and assembly, ECM-receptor interaction, actin cytoskeleton regulation, and the PI3K-Akt signaling pathway. These findings indicate that the 3D spheroids show enriched expression of gene programs associated with cell–matrix adhesion, focal adhesion signaling, and mechanotransduction, processes that are closely linked to bone maturation. The transcriptomic findings are consistent with observed mineralization patterns and prior literature, reinforcing the concept that 3D culture signaling enhances osteogenic differentiation via coordinated regulation of extracellular, cytoskeletal, and metabolic pathways.

Time-series analysis further refined our investigation, identifying 1522 genes with dynamic expression patterns. These DEGs were subsequently grouped into 8 clusters. We focused on cluster 2, 3, 6, and 7, which demonstrated significant upregulation, particularly under the 3D3W condition, termed the “3D-enhanced signaling.” Functional annotation of these clusters revealed a coordinated activation of programs not only for osteogenesis (“bone development,” “osteoblast differentiation”) but also for angiogenesis (“endothelial cell chemotaxis”) and immunomodulation (“leukocyte aggregation,” “positive regulation of inflammatory response”). The concurrent enrichment of these three functional axes is highly significant, as successful bone regeneration in vivo relies on the tightly coupled processes of osteogenesis [31], angiogenesis (to supply nutrients and oxygen) [32], and appropriate immune modulation (to coordinate the inflammatory phase and subsequent healing) [33]—a concept often referred to as osteo-angio-immune coupling. Subsequent PPI network analysis of the merged gene set from these clusters identified several key hub genes. Among them, FGF2 engaged in numerous interactions with other critical nodes, such as THBS1, NOTCH1, and TGFB3. The centrality of FGF2 suggests its potential role as a pleiotropic regulator, integrating signals across osteogenic, angiogenic, and immunomodulatory pathways to orchestrate the enhanced regenerative response observed in the 3D microenvironment.

## 5. Conclusions

Despite these insights, our study has several limitations. Firstly, high-resolution mitochondrial imaging in 3D spheroids remains technically challenging due to light scattering and dye penetration. Additionally, mitochondrial metabolic assays (OCR/ECAR, ROS) have not been performed on BMSCs under 2D and 3D culture conditions. Secondly, we did not perform profiling analysis of secreted factors (such as VEGF, TGF-β, cytokines) using ELISA and proteomics techniques, which could have strengthened the association between transcriptional features and paracrine outputs of the bone–vascular–immune axis. Finally, the functional roles of the identified hub genes, such as FGF2, in the 3D BMSC context remain to be experimentally confirmed through gain- or loss-of-function studies in future work.

## Figures and Tables

**Table 1:** The top five up- and down-regulated genes with the most significant differences.

Compare	Up-Regulated			Down-Regulated		
	Gene	p-adj	log2FC	Gene	p-adj	Log2FC
3D3W vs. 2D1W	*C3*	5.15 × 10^−169^	13.25	*Ccdc80*	1.61 × 10^−122^	−6.91
	*Lcn2*	2.81 × 10^−163^	13.41	*Itga11*	1.39 × 10^−89^	−7.60
	*Stc1*	1.61 × 10^−122^	7.37	*Mest*	3.81 × 10^−84^	−8.27
	*Serpina31*	5.44 × 10^−114^	12.82	*Acta2*	1.98 × 10^−81^	−7.30
	*Steap4*	9.59 × 10^−103^	7.70	*Cadm4*	8.59 × 10^−81^	−6.42
3D3W vs. 2D2W	*Slco4a1*	4.00 × 10^−86^	5.35	*Myh11*	3.40 × 10^−198^	−8.58
	*F5*	4.37 × 10^−48^	4.65	*Col14a1*	5.15 × 10^−124^	−7.79
	*Pla2g7*	2.56 × 10^−40^	4.48	*Acta2*	5.12 × 10^−121^	−8.06
	*Cd24*	1.01 × 10^−38^	6.03	*Smoc1*	1.92 × 10^−104^	−6.90
	*Serpinb8*	1.36 × 10^−34^	6.25	*Myl9*	5.07 × 10^−98^	−7.98
3D3W vs. 2D3W	*Slco4a1*	1.32 × 10^−64^	5.41	*Eln*	1.01 × 10^−176^	−11.18
	*Slc7a11*	1.36 × 10^−39^	4.49	*Myh11*	8.11 × 10^−170^	−9.35
	*Slc16a3*	3.36 × 10^−39^	3.54	*Acta2*	1.33 × 10^−114^	−8.72
	*Serpinb8*	4.85 × 10^−35^	5.17	*Smoc1*	3.41 × 10^−108^	−6.72
	*Meox1*	2.24 × 10^−34^	3.18	*Myl9*	3.80 × 10^−90^	−8.40

**Table 2:** The number of genes in each cluster.

Total Expressed Gene		Time Series Gene	
33,770		1522	
Cluster 1	Cluster 2	Cluster 3	Cluster 4
180	185	174	356
Cluster 5	Cluster 6	Cluster 7	Cluster 8
189	107	158	173

## Data Availability

The original contributions presented in this study are included in the article and Supplementary Material. Further inquiries can be directed to the corresponding author.

## Supplementary Materials

The following supporting information can be downloaded at: https://www.mdpi.com/article/10.3390/biom15111623/s1, Supplementary Video: 0 d, 7 d, 14 d, 21 d.
